# Cell Therapy for Androgenetic Alopecia: Elixir or Trick?

**DOI:** 10.1007/s12015-023-10532-2

**Published:** 2023-06-05

**Authors:** Yongcui Mao, Pinyan Liu, Jiayun Wei, Ye Xie, Qiuxia Zheng, Rui Li, Jia Yao

**Affiliations:** 1 The First Clinical Medical College of Lanzhou University, Lanzhou, China; 2 Key Laboratory of Biotherapy and Regenerative Medicine of Gansu Province, Lanzhou, China

**Keywords:** Androgenetic Alopecia, Stem Cells, Cells Therapy, Conditioned Media

## Abstract

Androgenetic alopecia is the most common cause of hair loss aggravated by increased life pressure, tension, and anxiety. Although androgenetic alopecia (AGA) does not significantly effect physical health, it can have serious negative impact on the mental health and quality of life of the patient. Currently, the effect of medical treatment for AGA is not idealistic, stem cell-based regenerative medicine has shown potential for hair regrowth and follicle repair, but the long-term effect and mechanism of stem cell therapy is not quite explicit. In this review, we summarize the methods, efficacy, mechanism, and clinical progress of stem cell therapies for AGA by now, hope it will present a more comprehensive view in this topic.

**Graphical Abstract:**

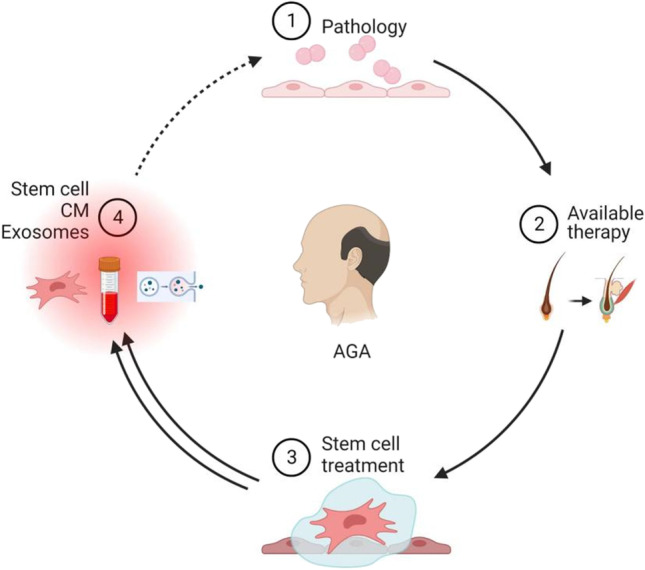

## Introduction

Alopecia is a globally prevalent disease which is mainly caused by genetics, hormonal disorders, immune inflammation, malnutrition, environmental factors, mental disorders, aging, and other factors [1]. Androgenetic alopecia (AGA), the most common type of hair loss, has emerged as a medical and social issue due to its onset at a young age. It can be related to psychological problems such as depression, anxiety, and mood disorders [2]. AGA is characterized by the progressive miniaturization of hair follicles and a shortened growth period of dermal papilla cells (DPCs) leading to hair loss [3]. By the age of 50, approximately 50% of men and 45% of women are affected by AGA [4]. Although the pathogenesis of AGA remains controversial, it is generally related with the expression of dihydrotestosterone (DHT), which is converted from free testosterone by type II 5-α reductase [5]. DHT miniaturizes hair follicles by gradual hair thinning during the growth period, shortens the hair growth cycle by thinning and shallowing the original coarse and black hair, and leads to hair loss due to hair follicle atrophy and extinction [6]. DHT accumulation in androgen-sensitive hair follicles results in a higher DHT expression in balding scalp tissues compared to the non-balding scalp tissues [7, 8]. AGA has been associated with the loss of attachment between an enlarged stem cell population and the arrector pili muscle [9].

**Fig. 1:**
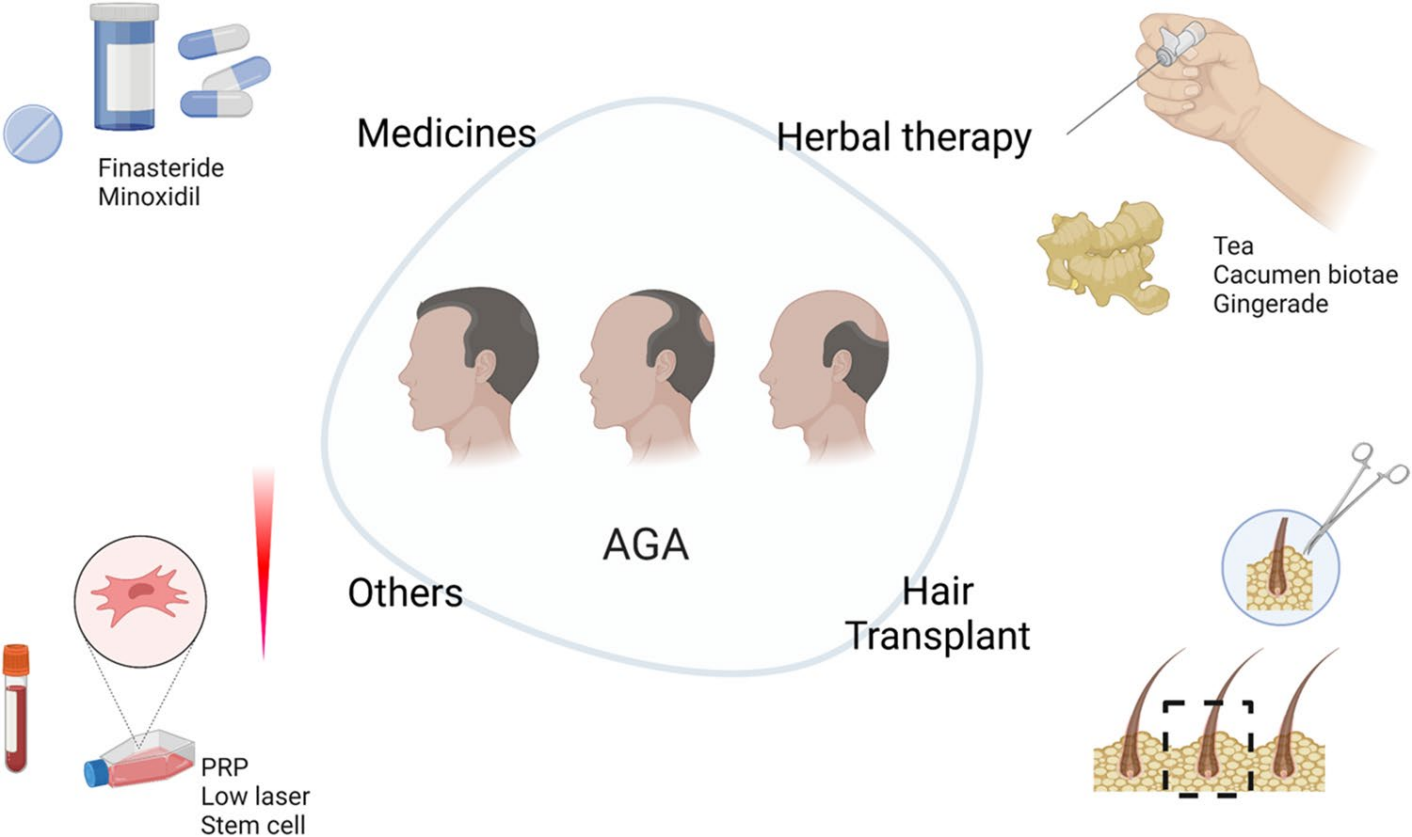
Traditional therapies for androgenetic alopecia

**Fig. 2:**
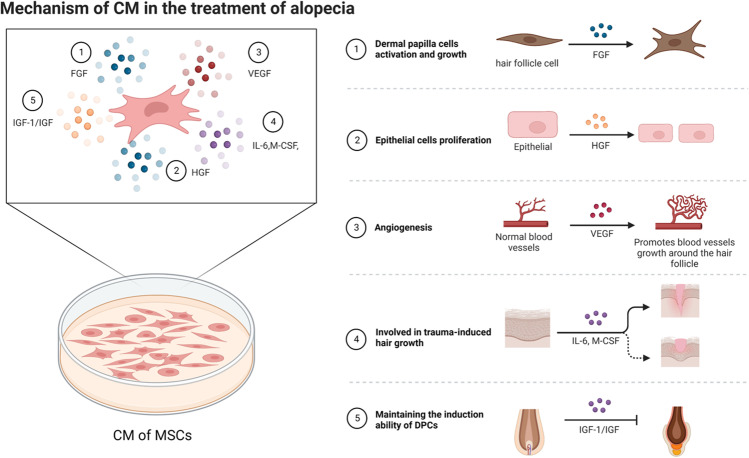
Mechanism of CM in the treatment of alopecia

**Fig. 3:**
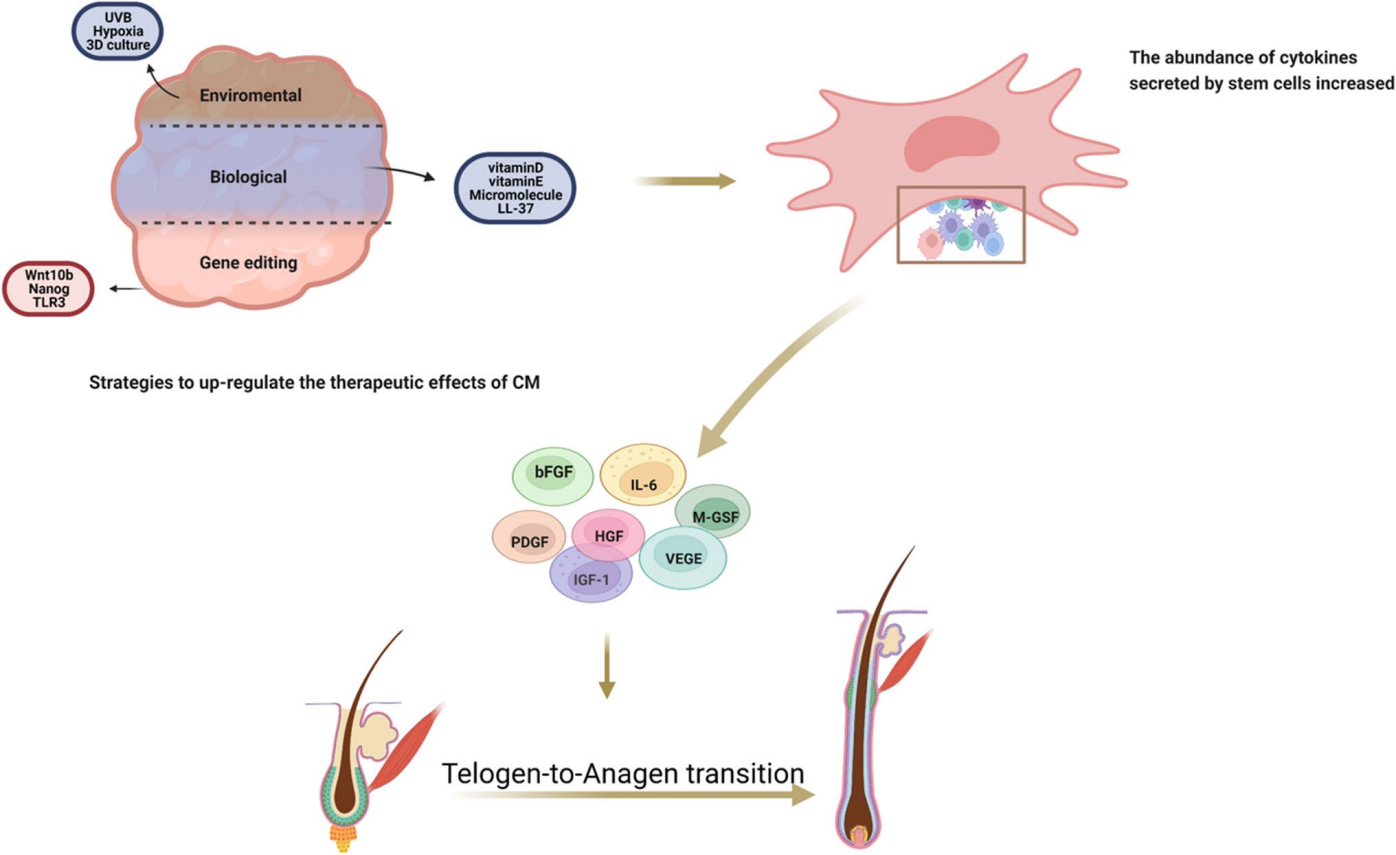
Strategies to up-regulate the therapeutic effects of CM on hair regeneration

At present, the treatments promoting hair regrowth or preventing hair loss include drug treatments, surgical treatments, herbal medicines, biotherapies, and other physical treatments [10]. Though effective, these treatments are limited and have several side effects [11].The treatment of hair loss using stem cells is not only cost effective but also produces faster results with simple treatment processes Fig 1, 2 and 3. As a result, stem cell therapeutics-related research has made significant achievements [12]. Stem cell technology has long been regarded as a "regenerative medicine technology" and has been hailed as the third medical revolution after drug therapy and surgery, Stem cell therapy has emerged as a new treatment for hair loss. This review provides a systematic review of the methods, efficacy, advantages and disadvantages of stem cell therapy for androgenetic alopecia from the perspective of the etiology of hair loss.

## Etiopathogenesis

### Genetics

The pathogenesis of androgenetic alopecia AGA is driven by androgens, with genetic susceptibility being the main prerequisite. Data have been reported showing that 35 of 57 childhood AGA patients (22 boys and 13 girls) had a family history, and 29 of the 35 (83%) had a family history of hereditary alopecia in a first or second degree relative[13]. A study of candidate genes and genome-wide associations analysed replicate sets containing 2759 cases and 2661 controls of European descent to confirm that the analysis identified four genome-wide significant risk loci for AGA on chromosomes 2q35, 3q25.1, 5q33.3 and 12p12.1. Of these, rs7349332 obtained the strongest association signal on chr2q35, which is located in WNT10A, providing genetic evidence for the involvement of WNT signalling in AGA development[14]. It has also been demonstrated that a variant of PADI3 is present in central centrifugal scarring alopecia [15]. The study of Rui et al. identified rs6493497 and rs7176005 as significantly associated with female pattern hair loss (FPHL) [16]. In addition, the authors found that rs4646 was not associated with FPHL,While rs4646 was one of the top ranked snp in the Yip et al.'s study[17]. Another study showed that susceptibility to early-onset FHPL was associated with the AR/EDA2R locus[18]. The study of Elaine G Y Chewet al identified differentially expressed genes between balding and non-balding DPCs, revealing downregulation of vascular-related genes in balding DPCs, evidence for AR rather than EDA2R as a candidate gene at the X-chromosome AGA risk locus.It also revealed that TWIST1 and SSPN are functionally relevant AGA genes at the 7p21.1 and 12p12.1 risk loci, respectively[19].

### Androgen/Androgen Receptor

Although genetic factors play an important role in androgenetic alopecia, an increasing number of studies suggest that androgen/androgen receptor is also one of the key mediators of AGA[20]. Hamilton's study of 21 pre-castration and post-castration adult males over 8 to 18 years showed no baldness after orchiectomy[21]. Castration before puberty prevents beard growth, while castration between 16 and 20 years partially prevents full beard development, and castration after 20 years has no effect on beard growth, suggesting a close association between AGA and androgens [22]. The survey found that androgens affect about 73% of men and 57% of women over the age of 80, and 58% of men over the age of 50 [23]. Androgens influence a variety of human skin functions through intracellular signalling pathways, such as sebaceous gland growth and differentiation, hair growth, epidermal barrier homeostasis and wound healing [24]. As AGA does not correlate with serum testosterone, free testosterone and bioavailable testosterone levels, its pathogenic basis may be mediated through intracellular signalling from hair follicle target cells [25]. Testosterone is a lipophilic hormone that is converted to the more potent DHT by the enzyme 5α reductase after freely penetrating the cell membrane into the cytoplasm, and is five times more potent than testosterone in binding to androgen receptors in susceptible scalp [26]. The effects of androgens such as testosterone and DHT on the skin are mainly mediated through the androgen receptor (AR), a ligand-dependent nuclear transcription factor belonging to the steroid hormone nuclear receptor superfamily [27]. Meanwhile, the androgen receptor is a polymeric complex that includes the heat shock proteins (HSP) HSP90, HSP70 and HSP56, initially located in the cytoplasm [28]. DHT is transferred to the nucleus after forming an AR-DHT dimer with an AR located in the cytoplasm of the target cell, and the AR co-activator is recruited to the AR-DHT complex, which binds to the androgen response element, leading to the transcription and eventual translation of the target gene into a protein that exerts biological activity [29]. Under the influence of androgens, the dermal papillae of the hair follicle secrete many cytokines such as TGFβ 1, IL-1α and TNFα, which induce premature termination of the early growth phase of the hair follicle [30]. Cortisone 17a-propionate (CB-03–01, also known as clascoterone, Breezula™) is a synthetic androgen receptor antagonist used to treat androgen-driven conditions, including acne vulgaris and AGA. one of the phase 2 studies (NCT02279823) compared CB-03–01 solution with 5% minoxidil solution and a control placebo for safety and efficacy in 95 male patients with AGA. After 6 months, the results showed an increase in the number of hairs in the target area in the CB-03–01 group compared to the control placebo group (12.7 vs. 2.9), but the 5% minoxidil solution was superior to both groups (18.8)[31].

### Microinflammation

Although AGA is classified as a non-inflammatory, non-scarring form of hair loss, histological evidence of inflammation has long been recognised [32]. A previous series of 17 women with AGA and 5 normal controls showed a significant association between the severity of inflammatory infiltration and the degree of miniaturisation [33]. In a study with a primary cohort of women with alopecia, biopsies revealed follicular shrinkage in all specimens and perifollicular lymphocytic infiltration in most specimens (87.9% AGA, 81.6% AA), with no significant difference in prevalence [34]. In close proximity to the hair follicle, there are subdermal TREM2 + macrophages called "Trichophages", uring the resting phase, Trichophages produce the cytokine OSM (Oncostatin M) which binds to the OSM receptor on hair follicle stem cells, phosphorylating STAT5 in hair follicle stem cells (HFSCs) and inhibiting stem cell activation, leading to prolonged resting phase and delaying hair growth[35]. Jaworsky examined the hair, transitional and alopecic scalp of three men and one woman with progressive alopecia; immunohistochemical results showed that thickening of the outer follicular sheath in the transitional and alopecic areas was associated with mast cell degranulation and fibroblast activation within the fibrous sheath; control biopsies did not show follicular inflammation, while the transitional area consistently showed an infiltration of activated T cells in the lower part of the follicular funnel [36]. A previous study by John Plante et al. showed that perifollicular infiltration was present in 73% of AGA and 84% of control specimens, however, funnel and isthmus involvement was more common in AGA (P < 0.037 and P < 0.012, respectively)[34]. In a study that identified inflammation-related regulators such as activator protein 1 (AP-1) subunits (FOS, FOSB, JUN, JUNB, etc.), TLRs, PTGS, EGRs, AREG, HSPA1B were substantially upregulated in the AGA group using transcriptome analysis at different locations in the hair follicle [37]. Further studies have demonstrated the important impact of Treg cells on the maintenance of HFSC and that immune cells are essential for hair regeneration [38]. These findings highlight the link between inflammation and hair loss [39].

### Others

Androgenetic alopecia is not only associated with genetics, immunity and microinflammation, but also with a number of other factors that influence the process of hair loss. Some in vitro human studies have shown increased markers of oxidative stress and increased sensitivity to oxidative stress in dermal papilla cells of balding scalp compared to non-balding scalp [40]. A study of microarray gene expression data from male patients with and without baldness on the scalp found that the expression of genes related to the oxidative stress pathway was upregulated in cultured human hair follicles and that oxidative stress has been shown to cause apoptosis and inhibit cell matrix growth [41]. Age is also strongly associated with AGA, with a decrease in HFSCs with age leading to hair loss [42]. Furthermore, it has been suggested that obesity is not only a risk factor for hair loss, but may synergize with repetitive hair cycles or aging-induced changes that significantly inhibit HFSC self-renewal [43]. Inhibition of autophagy with 3-methyladenine induces apoptosis, premature follicular regression and slows hair growth in organ culture follicles, so autophagic damage may be a potential mechanism for androgenetic alopecia [44]. A study that recruited 1,000 healthy men between the ages of 20 and 35 and developed a customised questionnaire to determine basic physical and smoking habits found that the prevalence of AGA was higher among smokers than non-smokers, and that the severity of AGA was not related to smoking intensity[45]. One questionnaire study found that age 30–40, marital status, poor sleep habits, meat consumption, junk food consumption, and heavy work were factors that influenced the development of more severe forms of AGA, another important finding of this study is that both late sleep and poor sleep quality also increase the risk of developing more severe types of AGA [46]. UV exposure also induces oxidative DNA damage and cytotoxicity in human hair follicle cells [47]. In addition, alopecia is also affected by microbiota. Hair follicles were extracted from the occipital and apical parts of alopecia patients and healthy volunteers, and 16S rRNA sequencing of the microbiome revealed that the middle hair compartment of the follicle was dominated by Burkholderia, and was less diverse; whereas bacterial diversity was higher in the lower part of the hair, with no significant differences between the occipital and apical follicles. In patients with alopecia, the lower middle compartment of the reduced hair tip contained Propionibacterium acnes, while non-reduced hair in other regions was comparable to healthy hair [48].

## Current Treatment for Hair Loss

For hair loss, early prevention, diagnosis, and treatment are internationally recognized. AGA shows progressive aggravation if left untreated. Therefore, AGA treatment aims to prevent hair miniaturization, induce hair thickening, and promote regrowth [49]. To date, finasteride and minoxidil are the only two FDA-approved drugs for the treatment of AGA [50]. Finasteride affects hair growth by acting on DPCs by improving the aggregation behavior of stem cells and enhancing the expression of stem cell transcription factors Nanog and Sox-2 [51]. However, long-term use of finasteride can negatively impact sexual functions, such as decreased libido, decreased ejaculation, increased infertility, and erectile dysfunction [52]. Minoxidil 5%, a common topical drug, when applied externally can stimulate DPCs and hair follicle stromal cells. It effectively prevents miniaturization of hair follicles and promotes limited regeneration [53]. However, the side effects include aggravation of seborrheic dermatitis, irritant contact dermatitis, and allergic contact dermatitis [54]. Bimatoprost, a PGF2 analog, has some hair growth inducing effects but is less effective than minoxidil [31].

Apart from the FDA-approved drugs, there are several treatments to combat hair loss. Some herbal medicines such as *Polygonum multiflorum*, *Astragalus membranaceus*, *Platycladus orientalis* leaves, and plum blossom needle puncture have similar curative effects [55, 56]. Their major drawback is that they have complex components, unclear mechanisms of action, and slow affects, which often requires long-term care to be effective [57]. Hair transplantation is another alternative but instead of increasing hair thickness it redistributes hair [58]. Interestingly, the transplantation surgery is considered invasive, and scarcity of hair for redistribution can be encountered [59]. Alternatively, microneedle therapy is a minimally invasive procedure that employs multiple fine needles to create microneedles on the skin and stimulate new blood vessels, Wnt protein expression, and growth factors [60]. When combined with other drugs, microneedles can further stimulate hair growth [61, 62]. Platelet-rich plasma can also treat AGA by improving the survival of DPCs during the hair growth cycle through anti-apoptotic effects [63, 64]. Low-dose light therapy not only improves the cure rate of AGA but also improves patient satisfaction [65]. Patients who do not respond to or choose medical or surgical treatment use hair patches, wigs, or scarves. The advantages and disadvantages of various treatment methods are compared in Table 1.

**Table 1:** Advantages and disadvantages of various treatments for androgenetic alopecia

Therapy method		Therapeutic effect	Advantage	Disadvantage	Reference
Surgical therapy	Hair Transplantation	Different degrees of shedding in the early postoperative period, no obvious effect can be seen until 6–9 months after the operation	Effective, Security	The high cost, Limited hair follicles	[66] [67]
	Microneedle	Hair density and diameter increased	Security, well tolerated	The pain is intense, a risk of infection	[68]
Drug therapy	Finasteride	Take orally 1 mg daily. Generally, hair loss decreases after 3 months	The incidence of adverse reactions was low and mild	Individual patients appear libido loss, impotence and ejaculation reduction, easy to relapse after drug withdrawal	[69]
	Minoxidil	Hair growth is significantly increased, hair loss is reduced, 5% formula is more effective	Effective promotion of hair regeneration, high safety	Dermatitis, headache and hypertrichosis as well as poor dependence, easy to relapse after drug withdrawal	[70]
Herbs therapy	Oral and Topical	Prevent and slow hair loss	High compliance, less side effects, wide spectrum of activity low price, wide range of use	Active ingredients are uncertain, plants and formulations are not standardized	[57]
Other therapy	Platelet plasma rich	After 6 months of subcutaneous injection, the average number and thickness of hair increased significantly	Minimally invasive, fewer safety issues and side effects	Preparation methods, frequency of treatment, and area of treatment have not been standardized	[71]
	Laser therapy	Promote hair regeneration alleviate hair loss, hair luster significantly improved	The laser comb is convenient and easy to operate	Transient hair loss during the resting period may occur at the beginning of treatment	[72]
	Stem cells	Promotes the proliferation of hair follicle cells	Economy, effective	Large, more robust double-blind controlled clinical trials are lacking to further evaluate the exact mechanism, therapeutic potential and safety of stem cell-based hair treatments	[12]

Current treatments for hair loss include drugs including finasteride and minoxidil, hair transplantation, platelet-rich plasma, low laser therapy and physical occlusion, ect.

## Stem Cell-based Therapies for AGA

With the development of regenerative medicine, stem cell-based therapies have received attention as potential new therapies. These therapies reactivate hair follicle stem cells, thereby promoting hair follicle growth, regeneration, and development [73, 74]. Stem cell therapy for AGA includes stem cell transplantation, stem cell-derived conditioned media, and stem cell-derived exosomes [12]. Compared to hair transplantation and drug therapy, stem cell-based biotherapy has brighter prospects. Table 2 lists some clinical trials involving stem cells for hair regeneration.

**Table 2:** Clinical trials based on stem cell in androgenetic alopecia

Stem cell source	Time	Recruitment	Method	Results	Reference
Adipose tissue	12 weeks	27	Application of AAPE™ with micro-needle roller	Hair density increased from 105.4 to 122.7 hairs/cm2 (P < 0.001); Hair thickness increased from 57.5 mum to 64.0 mum (P < 0.001)	[75]
Hair follicles	23 weeks	21	HFSCs injections 1 mL (0.2 mL·cm2) were administered to select areas of the scalp at a depth of 5 mm; Injections performed in two sessions spaced 60 days	29% ± 5% increase in hair density for the treated area and less than a 1% increase in hair density for the placebo area	[63]
Adipose tissue	16 weeks	38	130-mL topical solution, apply 2 mL of this solution to the hair loss area, twice every day for 16 weeks	Hair diameter after 16 weeks was observed in IG compared with that in CG, with the total percentage change from baseline of 14.2% vs 6.3%	[76]
Hair follicles	4 months	46	Apply one vial (5 mL) of the product on clean and dry scalp, apply every day for 5 consecutive days, stop the treatment for 2 days and then continue the application	After 2 and 4 months of treatment, the anagen rate was increased by 6.8% and 10.7%, respectively. Hair resistance to traction was decreased by 29.6% and 46.8%	[77]
Umbilical cord blood	3 months	15	The investigational product was transported frozen to the study site, thawed just before use, and applied using a 1.5 mm derma roller by the study investigator	Patient feedback demonstrated that 92% agreed that the investigational product was effective and were highly satisfied with the treatment after 3 months. Clinical evaluation indicated that 5–10% of young patients showed improvement in the control of graying of hair	[78]
Umbilical cord blood	24 weeks	87	Be directed to use on hair and scalp by subject her/himself at home twice a day (in the morning and evening) for 24 week	Not found	

## Stem Cell Transplantation

Stem cells are a class of self-replicating, multipotential undifferentiated cells, a relatively primitive class of cells that can differentiate into cells with multiple functions under certain conditions [79]. Stem cells can repair and renew damaged organs and tissues, activate self-healing, improve immunity, and reverse aging [80]. Therefore, stem cells have been widely used in the treatment of various diseases such as diabetes, myocardial infarction, Alzheimer's disease, and Parkinson's disease [81]. Additionally, stem cells are used in plastic surgery treatments, such as stem cell hair transplant [82]. The adipose tissue, bone marrow, hair follicles, and umbilical cord are the sources of regenerative pluripotent stem cells. Adipose-derived stem cells are used in skin anti-aging treatment due to their effective re-epithelialization, easy availability, and less discomfort to the patients [83]. Adipose interstitial vascular cells (ADSVCs) are rich in stem cells and adipose tissue; hence, can be used to treat hair loss [84]. Anderi et al. reported that all 20 patients with alopecia areata after receiving ADSVC treatment showed hair regeneration within 3–6 months [85]. A randomized, double-blind, drug-controlled trial in South Korea used adipose-derived stem cell component extract (ADSC-CE) for AGA, confirming the efficacy of treatment [6]. Zhang and Ye demonstrated that the epidermal stem cell transplant can induce hair follicle regeneration. They subcutaneously injected a mixture of epidermal stem cells and DPCs into nude mice. The injection site was stained with hematoxylin and eosin (HE) to observe hair follicles and epidermal regeneration [86]. Human umbilical cord mesenchymal stem cells (hUC-MSCs), a rich source of mesenchymal stem cell, have been promoted as a cell-based treatment option for tissue repair [87]. Bak et al. transplanted umbilical cord blood mesenchymal stem (UC-MSCs) cells into the C3H/HeJ mice [88]. After 5 weeks of treatment, diffused dorsal skin darkening was observed, with no significant change in the control group. This result demonstrated that the UC-MSCs blocked apoptosis and improved the number of hair follicles. Transplantation of human hair follicle mesenchymal stem cells (hHF-MSCs) into the scalp of 27 patients treated with placebo showed an increase in the density of hair in the target area by 18.0/0.65 cm^2^ and 23.3 /cm^2^, respectively, from baseline, while in the control group it reduced by 1.1/0.65 cm^2^ and 0.7/cm^2^ (P < 0.0001), after 58 weeks.The HF-SCs improved the hair density in the treated area by 5–29% in the 11 patients with AGA [89].

AGA has been effectively treated using stem cell transplantation, however, it faces cell therapy based regulatory and safety challenges, such as tumorigenesis and infection transmission issues [90]. Another approach is to utilize stem cell-based proteins and growth factors also known as stromal cell secretion groups or conditioned media (CM). This treatment uses a mixture of stem cells derived mesenchymal growth factors, microRNAs, and polypeptides [91].

### Stem Cell-derived Conditioned Media

The CM is rich in growth factors, cytokines, and beneficial proteins. In vitro and in vivo experiments that used hUC-MSCs for hair regeneration, confirmed the safety and effectiveness of CM in treating AGA [92]. CM has gained attention due to its easy collection and convenient transportation, and has broad prospects in regenerative medicine.

#### Applications of CM

Numerous studies and clinical trials have demonstrated the potential of CM for hair regeneration. The dermatology research team of Chung-Ang University School of Medicine in an experiment added transforming growth factor β1 (TGF-β1) protein and lithium chloride to the conditioned culture medium containing hUC-MSCs. After 24 h, the culture medium was replaced with the medium containing DPCs. After 3 days, the hUC-MCSs were collected from the conditioned culture medium. Subsequently, a 16-week clinical trial involving 30 patients with mild to moderate hair loss was conducted. The results indicated a significant increase in average hair density, hair thickness, and hair growth rate compared to the placebo group, with no adverse effects such as irritation or itching [93]. Similarly, a 3-month pilot study involving 15 volunteers using hUC-MSC-conditioned medium indicated that 86.6% of the volunteers had hair regeneration, with no side effects or adverse reactions [92]. Adipose-derived stem cell conditioning medium (ADSC-CM) too had similar effects. The results showed that after 12 weeks of ADSC-CM treatment in 27 female patients with alopecia, hair density increased from 105.4 to 122.7 roots/cm.^2^ (P < 0.001), and hair thickness increased from 57.5 μm to 64.0 μm (P < 0.001), with no occurrence of serious adverse reactions [75]. Based on the above results, an experiment was conducted in 40 patients for scalp regeneration. The patients were administered an intradermal injection of CM once a month for a period 6 months. The results proved that ADSC-CM not only promoted hair follicle regeneration but also promoted scalp regeneration between hair follicles [94]. Lee et al. locally applied ADSC-CM to 30 patients, who received non-ablative fractional laser treatment, and observed no adverse reactions. The experiment suggested that ADSC-CM can accelerate an increase in hair density and volume in patients with AGA after non-ablative fractal laser treatment [95]

#### Mechanism of CM in Treating Hair Loss

Stem cells use paracrine action of various growth factors and cytokines to perform their biological functions [96]. The CM derived from MSCs, a cell-free suspension rich in growth factors and cytokines, plays an important role in stimulating hair growth [97]. Cytokines secreted by stem cells in CM include vascular endothelial growth factor (VEGF), insulin-like growth factor (IGF), hepatocyte growth factor (HGF), platelet-derived growth factor (PDGF), bone morphogenetic proteins (BMPs), interleukin-6 (IL-6), and macrophage colony-stimulating factor (M-CSF) [98, 99]. VEGF protects CD200-rich and CD34-positive HFSCs from androgen-induced apoptosis via PI3K/Akt pathway. It reverses an increase in the Bcl-2/Bax ratio and a decrease in 5α-DHT induced increase in caspase-3 levels. To improve the hair follicle cycle, HGF promotes the growth of hair follicles by upregulating the expression of β-catenin [100], whereas IGF induces the proliferation and migration of MSCs by activating IGFR-mediated ERK1/2 signaling pathway [101]. Other experiments confirmed that PDGF and fibroblast growth factor 2 (FGF2) synergistically promote the proliferation of DPCs and enhance the expression of hair follicle-related genes to maintain their hair-inducing activity [102].BMPs maintained the DPC phenotype (the basis of HFSCs stimulation), whereas TGF-β regulated the hair-cycle signaling pathway, extracellular matrix synthesis, fibroblasts and mesenchymal stem cells proliferation, and hair follicles development [103].

#### Strategies to Upregulate the Regeneration Efficacy of CM

##### Environmental Stimulations

Byung. Park et al. demonstrated the effect of hypoxia on adipose-derived stem cells (ADSCs) stimulated hair growth. Hair regeneration induced by ADSC-CM cultured under hypoxic and normoxic conditions showed that hypoxia promoted hair growth in ADSCs. It significantly increased the secretion of insulin-like growth factor binding proteins (IGFBP-1 & IGFBP-2), M-CSF, M-CSF receptor, platelet-derived growth factor receptor-β (PDGFR-β), and VEGF [104]. ADSC-CM has a similar effect after 48 h of UVB irradiation. The passage of human skin fibroblasts, gradually decreased the relative expression of collagen I, collagen III, and elastin, whereas UVB irradiation decreased the expression of collagen I, collagen III, elastin, and TIMP-1 [105]. Moreover, after the same number of MSCs were inoculated into the 2D and 3D culture systems, the exosomes were isolated from medium supernatant and qualitative and quantitative analyses were conducted. The results showed that in 3D culture the surface markers of MSCs, and morphology and size of the 3D-Exos was unaltered. However, the total exosome production increased by 19.4 times in 3D culture compared with 2D culture. In the harvested supernatant, the level of 3D-Exos was 15.5 times higher than that of 2D-Exos. This suggested that changing the dimensions of the culture system also affects the therapeutic effect of CM [106]. Drzeniek N synthesized a 3D culture environment by encapsulating MSCs in collagen hyaluronic acid (col-HA) hydrogel. In-depth analysis of > 250 proteins in Col-HA-coated MSCs showed that the secretion spectrum of proangiogenic, neuroprotective, and immunomodulatory paracrine factors expanded.This enhanced the potential for angiogenesis [107].

##### Biological Factors

DPCs interact closely with the epidermal cells and play a key role in hair follicle induction and hair morphogenesis. DPCs often lose their ability to induce hair growth in in vitro monolayer culture, and it is difficult to obtain new hair follicle structures after transplantation in vivo. Therefore, small molecules such as SB431542 (SB, an inhibitor of the TGFβ/Smad pathway), CHIR99021 (CHIR, GSK3α/β inhibitor, and Wnt signal activator), and PGDF were added to the DPCs cultured with HaCaT CM. The mRNA expression of SOX2, ALP, and proteoglycans in DPCs were significantly upregulated [108]. In addition, vitamin d3 (VD3) pretreated CM significantly promoted hair growth. The experiments indicated that in preadipocytes pretreated with VD3, the VEGF level and angiogenesis rate was significantly increased in vivo than in in vitro [109]. A study on the signaling pathway indicated that ERK1/2 inhibitors reduced the production of VD3-enhanced VEGF, and the VD3 treatment increased ERK1/2 phosphorylation. Similarly, in vitamin E and selenium pretreated MSCs, the CD40 expression increased and the IL-12 expression decreased in MSC-conditioned medium. Hence, enhancing the ability of MSC to inhibit dendritic cells and enhance immune regulation [110]. LL-37, an antimicrobial peptide in the cathelicidin family, is widely found in the bone marrow, testis, neutrophils, monocytes, cervical, and vaginal squamous cells. LL-37 increased the expression of EGR1, activated MAPK, and the LL-37 pretreated ASCS-CM strongly promoted hair growth in vivo [111].

##### Gene Editing

Genetic modifications of stem cells can also enhance the expression of different growth factors. Wnt signaling pathway regulates hair morphogenesis and regeneration in embryos and adults. Adenovirus-mediated Wnt10b overexpression regulated the transition of hair follicles from the quiescent to the growth phase and induced hair follicle regeneration in mice [112]. Similarly, Wnt7a is associated with an increase in HFS at the wound site [113]. The generation of Wnt1A-CM by bone marrow mesenchymal stem cell (BM-MSC) accelerated the hair follicles from resting to growth stage, induced up-regulation of hair-related gene expression, and promoted the ability of DPCs to induce hair circulation and regeneration [112]. In addition, Nanog delayed senescence and maintained MSCs self-renewal ability in amniotic fluid source. Nanog expression of amniotic fluid source MSCs-CM, improved the secretion for hair regeneration and related factors, accelerated the transformation of hair follicles from the stationary to growth phase, and increased the density of HF [114]. In TLR3 agonist poly (I: C) pretreated bone marrow-derived MSCs, the antibacterial and immunomodulatory proteomic profiles of EV secretion were enhanced but the exosomes (EV) miRNA level remained unchanged [115].

Change cell culture conditions (such as 3D culture, low oxygen, ultraviolet irradiation, etc.) or with some small biological molecules training (such as vitamin D, antimicrobial peptide LL-37 etc.) or by means of genetic engineering of cells for editing strategy can increase the expression of effective components in conditioned medium quantity to increase, which affects the final pretreatment can increase the therapeutic effect of stem cells.

### Stem Cell-derived Exosomes and Their Applications

EVs are small particles of 30–1000 nm diameter. They are double coated with phospholipids and contain DNA, RNA, and proteins [116]. EVs enhance tissue regeneration, participate in immune regulation, serve as a carrier for therapeutic drugs, and are a potential alternative to stem cell therapy [117], 118. The stem cell action is mainly paracrine action mediated by stem cell secretory factors [88]. EVs are important components of stem cell secretion that are abundant in body fluids and can be released into the cell medium (CCM) [119]. Derivative EVs can promote the anagen phase and delay the catagen phase. Co-culture of DPCs and HFSCs induced differentiation of HFSCs. DPC-Exos attached to HFSCs surface, regulated proliferation and differentiation of HFSCs through cell signal transduction genes, regulated fatty acid expression, and cell communication [120]. Inflammatory human pulp stem cell-derived EVs have the potential to promote angiogenesis [121]. Adipose-derived EVs reduced inflammation and promoted wound healing [122]. In addition, EVs improve aging and age-related diseases; however, the underlying mechanisms are not known. Conversely, aging also affected the generation rate and loading of mesenchymal stem cells and their EVs [123]. EVs have advantages in the treatment of hair regeneration, such as direct fusion with the target cells, biological effects, long-term storage, and transportation at -70℃. Additionally, the concentration, dosage, route, and time of medication are easy to control, with no risk of immune rejection and tumor occurrence caused by cell transplantation therapy [124]. The amount of EVs present in CM is not high, and the methods that balance efficiency and purity of EVs in CM have not been developed [125]. Additionally, EVs have the potential risk of uncontrolled genetic information, immune response, and biological distribution. Cell-derived EVs have the potential to promote angiogenesis in vitro [126]. A comparison of stem cells, CM, and EVs for hair regeneration is presented in Table 3.

**Table3:** Comparison of stem cell, CM and exosomes for hair regeneration

Therapy method	Strengths	Weaknesses
Stem cell transplantation	Low immunogenicity[127] Multidirectional differentiation [128] Easy differentiation into various cell lines, and high angiogenesis potential[129]	Oncogenic[128]Infectious transmission, strict ethical review and high cost[130]
CM	Low tumor risk and cost of collection[131]easy preparation[128]	Short half-life and depletion of paracrine factors may require extensive and frequent administration [131]Lack of standardization of therapy[132]
Exosome	Avoid the degradation of effective active ingredients[133] Stable mass production[134]	Lack of effective separation methods[135], standardized guidelines for mass production and potential biosafety tissues[133]

## Challenge and Frontier

Stem cell-based treatments for hair loss have become popular in recent years. Although stem cell transplantation, CM, and EV therapies have been successful in preclinical and clinical stages, each therapy has its own limitations. As the stem cell transplants are expensive and tumorigenic, CM, and EVs may be more economical and safer for treating hair loss, though both have their own limitations. Due to the cell-free nature of CM, it is safer and more immunologically compatible, but isolating compositionally consistent CM is a challenge. Similarly, using EVs for treatment has its own challenges, where based on the intended therapeutic use, it is essential to select and characterize cell sources suitable for EVs production. Another important aspect is the method and conditions of cell culture as they have an impact on EVs yield, stability, and storage. Off-the-shelf therapies need further exploration. In addition, exosome isolation and storage methods lack standardization. The characterization of therapeutic exosomes should be further validated in relevant preclinical models to assess safety/toxicology, pharmacokinetics, and pharmacodynamics. To increase the likelihood of clinical translation, standardisation of stem cell culture, collection, preservation and validation of CM and isolation of exosomes is inevitable. Although the results are promising and robust, double-blind controlled clinical trials are needed to further evaluate the exact mechanism, therapeutic potential, and safety of stem cell-based hair loss management.

## Conclusion

The rapid development of regenerative medicine provides new ideas and directions for the treatment of AGA. Although, efficacy of stem cell transplantation in hair regeneration is encouraging, the biosafety of stem cells may hinder its clinical application. Owing to the secretions of stem cells, tumorigenic problems of stem cell transplantation have been eliminated. Therefore, there has been an increased interest in stem cell-conditioned media, exosomes, and related therapies. Available experimental studies have shown that both stem cells and stem cell-based non-cellular therapies can promote hair regeneration and prevent hair loss. The convenience, safety, effectiveness, ease of treatment and patient acceptance of stem cell therapy over medication and hair transplantation offer an alternative treatment option for hair loss patients.

## Data Availability

The datasets analyzed in this study are available from the corresponding author on reasonable request.
